# Low-Molecular-Weight Heparin-Functionalized Chitosan-Chondroitin Sulfate Hydrogels for Controlled Release of TGF-β3 and *in vitro* Neocartilage Formation

**DOI:** 10.3389/fchem.2019.00745

**Published:** 2019-11-01

**Authors:** You-Rong Chen, Zhu-Xing Zhou, Ji-Ying Zhang, Fu-Zhen Yuan, Bing-Bing Xu, Jian Guan, Chao Han, Dong Jiang, Yan-Yu Yang, Jia-Kuo Yu

**Affiliations:** ^1^Knee Surgery Department of the Institute of Sports Medicine, Peking University Third Hospital, Beijing, China; ^2^School of Clinical Medicine, Weifang Medical University, Weifang, China; ^3^Beijing National Laboratory for Molecular Sciences, State Key Laboratory of Polymer Physics & Chemistry, Institute of Chemistry, Chinese Academy of Sciences, Beijing, China; ^4^College of Materials Science and Engineering, Zhengzhou University, Zhengzhou, China

**Keywords:** low-molecular-weight heparin, hydrogel, controlled release, peripheral blood mesenchymal stem cell, tissue-engineered cartilage

## Abstract

Repair of hyaline cartilage remains a huge challenge in clinic because of the avascular and aneural characteristics and the paucity of endogenous repair cells. Recently, tissue engineering technique, possessing unique capacity of repairing large tissue defects, avoiding donor complications and two-stage invasive surgical procedures, has been developed a promising therapeutic strategy for cartilage injury. In this study, we incorporated low-molecular-weight heparin (LMWH) into carboxymethyl chitosan-oxidized chondroitin sulfate (CMC-OCS) hydrogel for loading transforming growth factor-β3 (TGF-β3) as matrix of peripheral blood mesenchymal stem cells (PB-MSCs) to construct tissue-engineered cartilage. Meanwhile, three control hydrogels with or without LMWH and/or TGF-β3 were also prepared. The gelling time, microstructures, mechanical properties, degradation rate, cytotoxicity, and the release of TGF-β3 of different hydrogels were investigated. *In vitro* experiments evaluated the tri-lineage differentiation potential of PB-MSCs, combined with the proliferation, distribution, viability, morphology, and chondrogenic differentiation. Compared with non-LMWH-hydrogels, LMWH-hydrogels (LMWH-CMC-OCS-TGF-β3) have shorter gelling time, higher mechanical strength, slower degradation rate and more stable and lasting release of TGF-β3. After two weeks of culture *in vitro*, expression of cartilage-specific genes collagen type-2 (COL-2) and aggrecan (AGC), and secretion of glycosaminoglycan (GAG), and COL-2 proteins in LMWH-CMC-OCS-TGF-β3 group were significantly higher than those in other groups. COL-2 immunofluorescence staining showed that the proportion of COL-2 positive cells and immunofluorescence intensity in LMWH-CMC-OCS-TGF-β3 hydrogel were significantly higher than those in other groups. The LMWH-CMC-OCS-TGF-β3 hydrogel can slowly release TGF-β3 in a long term, and meanwhile the hydrogel can provide a biocompatible microenvironment for the growth and chondrogenic differentiation of PB-MSCs. Thus, LMWH functionalized CMC-OCS hydrogels proposed in this work will be beneficial for constructing functional scaffolds for tissue-engineered cartilage.

## Introduction

Cartilage injury has brought about an increasing social and economic burden as a common joint disease (Everhart et al., 2019). On account of its avascular characteristics and lack of endogenous repair cells, articular cartilage has a limited regenerative and self-healing ability (Armiento et al., 2018). Currently, traditional clinical methods included the osteochondral transplantation, bone marrow stimulation and autologous chondrocyte transplantation, but they still could not achieve the satisfactory therapeutic effect (Redondo et al., 2018). Fortunately, tissue engineering technology has provided a promising therapeutic strategy for cartilage injury (Liaw et al., 2018; Zylinska et al., 2018; Ding et al., 2019). By means of the three elements of tissue engineering methods (biological scaffolds, growth factors, and seed cells), tissue-engineering cartilage possesses unique capacity to repair large tissue defects, avoid donor complications and two-stage invasive surgical procedures and has a wide range of scaffold materials and seed cells sources (Andriolo et al., 2017; Sadlik et al., 2017; Kwon et al., 2019).

For development of desirable tissue engineering program, it is urgent to prepare the functionally specific biomaterials that mimic the components of natural extracellular matrix of cartilage for biomedical applications (Stuckensen et al., 2018). As a typical biological scaffold, hydrogel has been widely utilized on account of their highly hydrated three-dimensional cross-linked soft-wet materials with good biocompatibility (Pan et al., 2013; Liu and Hsu, 2018; Hu et al., 2019). As for construction of tissue-engineered cartilage, hydrogel can provide a suitable microenvironment for cartilage differentiation, and cartilage-specific extracellular matrix (ECM) regeneration (Wang et al., 2019; Zhang Y. et al., 2019). Heparin is a kind of highly sulfated anionic glycosaminoglycan existing in extracellular matrix, which can bind electropositive protein and growth factors into stable complexes by electrostatic interaction to repair and regenerate various tissues (Sun et al., 2018; Thones et al., 2019). Heparin-functionalized hydrogel scaffold can protect proteins or growth factors from degradation and maintain their biological activity *in vivo*, meanwhile the electrostatic interaction can effectively avoid burst release and realize the sustained release of proteins or growth factors (Kim I. et al., 2018; Kim S. et al., 2018). Compared with unfractionated heparin (UFH), low-molecular-weight heparin (LMWH) has superiority on the long half-life, less bleeding side effects and no need for laboratory monitoring (Ali-Hassan-Sayegh et al., 2016; Robertson and Jones, 2017). Therefore, LMWH-functionalized hydrogel scaffold may be a kind of suitable and promising materials for generating tissue-engineered cartilage.

**Figure 1:**
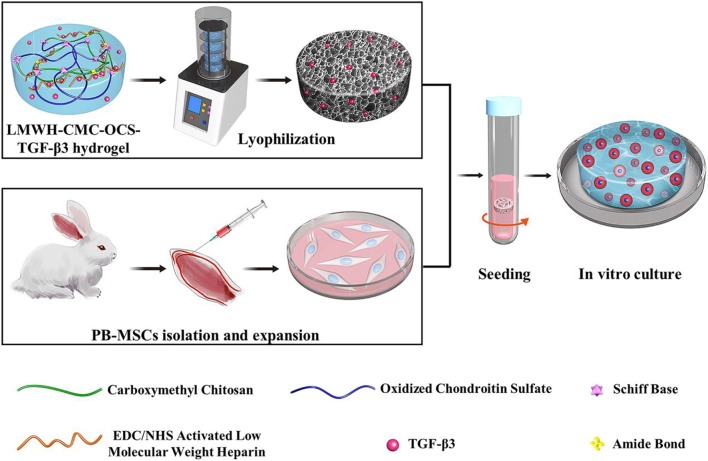
Schematic illustration of LMWH-CMC-OCS-TGF-β3 hydrogel as matrix of PBMSCs for constructing tissue-engineered cartilage *in vitro*.

Incorporation and controlled release of bioactive factor is a universal strategy to promote tissue repair and regeneration (Li X. et al., 2016; Qian et al., 2017). During the process of cartilage repair and regeneration, transforming growth factor-β3 (TGF-β3), as one of the relatively newer isoforms of TGF-β, can effectively promote the differentiation of mesenchymal stem cells (MSCs) into cartilage cell and induce the expression of extracellular matrix of cartilage by regulating the metabolism of articular cartilage and multifunctional proteins in a time-and concentration-dependent manner (Crecente-Campo et al., 2017; Wang et al., 2017). TGF-β3 can inhibit the activity of inflammatory mediators such as IL-1, MMPs, and TNF-α, and meanwhile reduce the body's immune response (Yanagawa et al., 2016; Frangogiannis, 2017). In addition, TGF-β3 plays a vital role in the growth and reconstruction of cartilage in both *vitro* and *vivo*, which has been proved by many previous reports (Yang et al., 2017; Deng et al., 2019). MSCs are multipotent precursor cells with multidirectional differentiation, self-renewal and low immunogenicity, which are widely used as seed cells for cartilage repair and regeneration (Liu H. et al., 2018; Han et al., 2019). Compared to frequently-used bone marrow mesenchymal stem cells (BM-MSCs), peripheral blood mesenchymal stem cells (PB-MSCs) possess distinct advantages, for example more minimally invasive, less complications (Wang et al., 2016a). Our previous studies proved that PB-MSCs had similar biological characteristics to BM-MSCs and even better cartilage differentiation tendency in each of monolayer culture and three-dimensional condition (Fu et al., 2011; Wang et al., 2016a). Therefore, PB-MSCs are one better kind of seed cells for repair and regeneration of bone and cartilage *in vivo* (Fu et al., 2014). In this work, we prepared a low-molecular-weight heparin-functionalized chitosan-chondroitin sulfate hydrogel, which could load TGF-β3 as matrix of PB-MSCs to construct tissue-engineered cartilage (Figure 1). The hydrogel scaffold can be capable of mimicking the biochemical composition of cartilage tissue and providing a bionic environment for seed cells; in this case, the TGF-β3 can be controlled released from the hydrogel in about one month and effectively promote differentiation into cartilage of PB-MSCs. This study will open a new venue for fabricating tissue scaffolds materials and investigating the mechanism/behavior of cartilage differentiation from PB-MSCs.

## Materials and Methods

### Materials

Chondroitin sulfate (CS, *M*n = 19.9 kDa) was provided by Xingyuan Chemical Reagent Co., Henan. Carboxymethyl chitosan (viscosity of 10 ~ 80 mPa·s, carboxylation degree of 80%) and low-molecular-weight heparin (LMWH, *M*n = 5,600–6,400 Da) were obtained from Dalian Meilun Biotechnology Co., Ltd. Dalian, China. Sodium periodate, 1-ethyl-3-(dimethylaminopropyl) carbodiimide hydrochloride (EDC) and N-hydroxysuccinimide (NHS) were purchased from Sinopharm Chemical Reagent Co., Ltd. Shanghai, China. Recombinant Human TGF-β3 was obtained from PeproTech, Inc. USA. TGF-β3 ELISA kit was purchased from Cloud-Clone Corp, China.

### Preparation of Oxidized Chondroitin Sulfate (OCS) and EDC/NHS-activated LMWH

Five gram of CS was reacted with 3.5 g of NaIO_4_ in 100 mL of distilled water in dark for 6 h, and then OCS was separated by adding 200 mL alcohol to the mixture and stirring for another 15 min (Fan et al., 2017). LMWH solution was prepared at a concentration of 1 mg/mL in PBS (0.01 M, pH 7.4), and carboxylic acid groups of LMWH (LMWH-COOH) were activated using NHS groups at a molar ratio of 10:10:1 (EDC: NHS: LMWH-COOH). The reaction was allowed to proceed for 12 h at 37°C with a pH value of 5.7 (Elahi et al., 2014; Liu Z. et al., 2018). The OCS and EDC/NHS activated LMWH were dialysis against the distilled water for 72 h and further lyophilized to obtain a purified material.

### Preparation of Hydrogels

Solutions of CMC (30 mg/mL) and OCS (100 mg/mL) were mixed with each other (volume ratio = 4: 1) in room temperature to obtain the CMC-OCS hydrogel. To prepare CMC-OCS-TGF-β3, LMWH-CMC-OCS and LMWH-CMC-OCS-TGF-β3 hydrogels, EDC/NHS-activated LMWH, and/or TGF-β3 were added to the OCS solution before mixing with CMC solution. EDC/NHS-activated LMWH and/or TGF-β3 were added within hydrogels at concentrations of 1.5 mg/mL and/or 250 ng/mL, respectively. The loading contents of TGF-β3 in hydrogels of CMC-OCS-TGF-β3 and LMWH-CMC-OCS-TGF-β3 were 5.68 and 5.49 ng/mg, respectively. The gelation time of hydrogel was obtained by the vial tilting method (Bu et al., 2016) and rheological measurements (time sweep experiment).

### Fourier Transform Infrared Spectra

Hydrogels were lyophilized in freeze dryer at −80°C for 48 h. The film samples of CS, OCS, CMC, LMWH, activated LMWH, LMWH-CMC, CMC-OCS hydrogels, and LMWH-CMC-OCS hydrogels were prepared by the technique of potassium bromide tableting. TENSOR-27 spectrometer (Bruker) was used to obtain the Fourier transform infrared (FTIR) spectra of samples with the method of previous reported (Bu et al., 2017).

### Scanning Electron Microscopy

A JSM-7900F scanning electron microscope (SEM, Hitachi, Japan) was used to observe the microstructure of the hydrogels. Briefly, the freeze-dried hydrogels were sliced and bonded to the sample stage with conductive resin, and then coated with a layer of gold and obtain the SEM images at 3 kV acceleration voltage. The pore size of hydrogels was measured with the help of Image-pro^®^ Plus 6.0 (Media Cybernetics, Inc., Rockville, USA) (Yang et al., 2018b).

### Porosity Determination

The method of liquid displacement was used to determine the porosities of freeze-dried hydrogels. First, samples with regular form were calculated and weighed the initial volume and weight (V_0_ and W_0_). Second, samples hydrogels saturated with absolute ethyl alcohol were weighted again (W_1_). The equation as below was used to calculate the porosities of four hydrogels (Qin et al., 2018):

$$\begin{array}{l} \text{Porosity }\left(\text{\%}\right)=\;\left[\left({\text{W}}_{1}-{\text{W}}_{0}\right)/\left({\text{V}}_{0}\times \text{ρ}\right)\right]\times 100\text{\%} \end{array}$$

ρ represents the density of absolute ethyl alcohol.

### Rheological Measurements

Thermo Haake Rheometer with a cone-parallel plate geometry (d = 35 mm) was used to measure the gelation time and shear modulus of four hydrogels. Time sweeps experiments were performed with a constant strain of 0.05% and oscillatory frequency of 1 rad/s. The samples in solution state (0.5 mL) were placed between two plates and the gap was set at 0.5 mm. Frequency sweep experiment were performed with a constant strain of 0.05% in the frequency range of 0.1–10 rad s^−1^. The hydrogels (d = 35 mm, h = 3.5 mm) were placed between two plates and tested at a gap of 3 mm (Wu et al., 2010; Wang L. et al., 2016).

### Compressive Testing

The mechanical properties of hydrogels were calculated with a universal material testing machine of Instron 3365 (Instron Co., USA). Samples were prepared (diameter 15 mm and height 7.5 mm) and compressive testing was carried out with a rate of 3 mm/min. The fracture strain, fracture compressive stress and compressive elastic modulus were calculated based on the stress-strain curve. Every kind of hydrogels was tested three times (Yang et al., 2016).

### Swelling Properties

The swelling properties of hydrogels were measured according to the methods reported in previous literature (Bu et al., 2016). We prepared the dried hydrogel samples (20 mg) and recorded the initial weight (W_d_), and then placed samples in 50 mL of PBS (pH 7.4) at 37°C. The samples were weighed again (W_s_) after incubation for certain time (0.25, 0.5, 1, 2, 4, 6, 8, 12, 16, 24, 48, 72, 96, 120, 144, and 168 h). The calculational equation of swelling ratio was used as below:

$$\begin{array}{l} \text{Swelling ratio }\left(\text{\%}\right)\;=\left({\text{W}}_{\text{s}}-{\text{W}}_{\text{d}}\right)/{\text{W}}_{\text{d}}\times 100\text{\%} \end{array}$$

### Degradation *in vitro*

The weight loss of samples in PBS solution during 21 days were recorded to describe the degradation behaviors of hydrogels. We recorded the initial weight of lyophilized samples (W_0_), and incubated them in PBS solution at 37°C. After incubating for certain time (1, 4, 7, 14, and 21 days), the samples were lyophilized and weighed again (W_t_). The degradation ratio can be obtained by the following equation:

$$\begin{array}{l} \text{Degradation ratio }\left(\text{\%}\right)=\;\left({\text{W}}_{0}-{\text{W}}_{\text{t}}\right)/{\text{W}}_{0}\times 100\text{\%} \end{array}$$

### TGF-β3 Release Experiments

The lyophilized samples (4.4 mg, *n* = 5) were placed in PBS solution (pH 7.4, 1 mL) at 37°C under the continuous agitation to obtain the release behavior of TGF-β3. After incubation for certain time (1, 4, 7, 10, 14, and 21 days), we withdrawn the PBS solution with released TGF-β3 and added same volume of fresh PBS to maintain the total volume of 1 mL. The released amounts of TGF-β3 was quantified with sandwich enzyme-linked immunosorbent assay (ELISA) using a Human TGF-β3 ELISA Kits (Cloud-Clone, Corp., Houston, TX, USA) (Ariyati et al., 2019).

### Isolation and Culture of PB-MSCs

The Animal Care and Use Committee of Peking University Third Hospital approved all of the protocols of animal experiments which were implemented follow the Guide for the Care and Use of Laboratory Animals. Peripheral blood (PB, 20 mL) was isolated from the central auricular arteries of New Zealand White rabbits after mobilizing with granulocyte colony stimulating factor (G-CSF, Qilu Pharmaceutical Co. Ltd.) and AMD3100 (MedChemExpress LLC., USA). Peripheral blood mononuclear cells (MNCs) were collected by using the method of density gradient centrifugation, and cultured in medium of α-MEM with 15% fetal bovine serum, 100 U/mL penicillin and 100 U/mL streptomycin. Culture medium was replaced every 3 days until the confluence of primary PB-MSCs reached around 90%, and then subculture was carried out at ratio of 1:3. PB-MSCs at passage 3 [PB-MSCs (P3)] were used for subsequent experiments (Fu et al., 2011).

### Tri-lineage Differentiation Potential

Multilineage differentiation potential of PB-MSCs was assessed with the method of tri-lineage differentiations. Osteogenic medium, adipogenic induction and maintenance medium, and chondrogenic medium (Cyagen Biosciences Inc., Suzhou, China), following the manufacturer's instructions, were used for osteogenesis, adipogenesis, and chondrogenesis of PB-MSCs, respectively. The deposition of calcium nodules, accumulation of lipid vacuoles in cells, and cartilage-specific aggregating proteoglycans were detected with the methods of Alizarin red staining, Oil red O staining, and Alcian blue staining (Fu et al., 2011).

### Cytotoxicity Studies of Hydrogels

Cell Counting Kit-8 assay (CCK-8, Dojindo Laboratories, Japan) was applied to evaluate the cytotoxicity of hydrogel by culturing PB-MSCs with the extracting liquid of hydrogels. Rabbit PB-MSCs at passage 3 were seeded into 96-well microplates (2 × 10^3^ cells/100 μL/well), and then 10 μL of hydrogel extracting liquid or PBS was added into cell medium and further incubated for predetermined time. After incubation for 12, 24, and 48 h, we removed the original culture medium and added fresh culture medium (100 μL) with CCK-8 reagent (10 μL). A microplate reader (Thermo, USA) was used to obtain the optical density (OD) value of 450 nm after incubating for another 1 h. The equation as below was used to calculate the cell viability:

$$\begin{array}{l} \text{Cell viability}=\left[\left(\text{As}-\text{Ab}\right)/\left(\text{Ac}-\text{Ab}\right)\right]\;\times 100\text{\%} \end{array}$$

Where the As, Ac, Ab are the optical density (OD) of hydrogels for the extracts group, control group and blank group. It was considered to be cytotoxic if cell viability was <70% after incubation with hydrogel extracting liquid (Bu et al., 2017).

### Cell Seeding and Cell-Scaffold Construct Culture

Fifty microliter of PB-MSCs suspension (1 × 107 cells/mL) was carefully seeded on freeze-dried hydrogel (diameter of 6 mm and thickness of 2 mm) with the method of centrifugation as we previously reported (Zhang Z. et al., 2015). The cell-hydrogel composites were incubated for 1 h to facilitate cell adhesion, and then 2 mL fresh chondrogenic differentiation medium (removal of TGF-β3 components, Cyagen Biosciences Inc., Suzhou, China) was added for further culture. Culture medium was replaced every 3 days until cell-hydrogel composites were used in subsequent experiments.

### Cell Distribution on Scaffolds

LIVE/DEAD Viability/Cytotoxicity Kit assay (Invitrogen, CA, USA) was used to visualize the cell survival and distribution on hydrogels. After culturing for 7 days, the cell-hydrogel composites were washed with PBS solution to remove culture medium, and immersed in reagents of calcein AM (2 mM) and ethidium homodimer-1 (4 mM) for 1 h at 37°C. Live (green) and dead (red) cells was detected by using a confocal microscopy with excitation wavelength of 568 and 488 nm. Imaris software 7.4.2 (Bitplane, Oxford) was used to create three-dimensional rendering in order to observe and analyze the distribution of PB-MSCs on hydrogels.

### Cell Proliferation on Hydrogels

Cell proliferation on hydrogels was assessed by using the method of CCK-8 assay. After incubation for 1, 3, 5, and 7 days, we removed the original culture medium, and added fresh culture medium (100 μL) with CCK-8 reagent (10 μL). A microplate reader (Thermo, USA) was used to obtain the optical density (OD) value of 450 nm after incubating for another 2 h. The cell proliferation curves of each groups obtained by normalizing the OD value at each point against the average value of the first day.

### Analysis of Cartilage-Specific and Hypertrophic Genes Expression

After incubation for 7 and 14 days, cell-hydrogel composites were removed from culture medium and washed with PBS. TRIZOL reagent (Invitrogen, Carlsbad, CA, USA) and RevertAid First Strand cDNA Synthesis Kit (K1622, Thermo Scientific, Carlsbad, CA, USA), following the manufacturer's instructions, were used to extract total RNA and reverse-transcribe isolated RNA, respectively. According to the conditions reported in previous literature (Zhang Z. Z. et al., 2019), quantitative Real-time polymerase chain reaction (RT-PCR) analysis was performed to detect the expression of cartilage-specific marker gene (COL-2 and aggrecan, AGC) and hypertrophic marker gene (collagen type-10, COL-10) by using an ABI 7300 real-time PCR system (Applied Biosystems, Foster City, CA, USA) with SYBR^®^ Select Master Mix (4472908, Thermo Scientific, Carlsbad, CA, USA). The value of relative expression in these target genes were plotted as 2^−ΔΔCT^ with the method of previous reported (Zhang Z. Z. et al., 2019). The PCR primers are listed in Table S1.

### Quantification of DNA, Collagen Type-2 (COL-2), and Glycosaminoglycan (GAG) Content

Hoechst33258 staining and fluorometric assay was performed to measure the DNA content of cell-hydrogel composites. After culturing for 1, 7, and 14 days, the cell-hydrogel composites were weighed and then digested in a prepared papain solution (Sigma, St. Louis, Missouri, USA) at 60°C for 24 h to obtained aliquots of the sample digestion. Aliquots of the sample digestion (10 μL) were mixed with Hoechst33258 working solution (2 μg/mL, 100 μL) and incubated at 37°C for 1 h. A microplate reader (Thermo, USA) was used to detect the fluorescent intensities with excitation wavelength of 360 nm and emission wavelength of 460 nm. A standard curve of calf thymus DNA (Sigma, St. Louis, Missouri, USA) was used to normalize the content of DNA.

High efficiency RIPA tissue/cell lysis solution (R0010, Solarbio Science & Technology Co., Ltd., Beijing, China) was used to obtain total proteins of the cell-hydrogel constructs. Rabbit COL-2 ELISA Kits (Cloud-Clone, Corp., Houston, TX, USA) and Rabbit GAGs ELISA kit (BlueGene Biotech., Shanghai, China) were applied to measure the COL-2 content according to the manufacture protocol, and the contents of COL-2 and GAG were normalized to DNA content.

### Immunofluorescent Staining of COL-2

Immunofluorescent staining was performed to visualize the secretion of COL-2 in cell-hydrogel composites after culturing for 14 days. First, cell-hydrogel composites were washed with PBS to remove culture medium and fixed with 4% paraformaldehyde. Then, 10% bovine serum albumin (BSA), mouse anti-COL-2 primary antibody (CP18, Merck KGaA, Darmstadt, Germany), Alexa Fluor^®^ 594 goat anti-mouse IgG antibodies (ZF-0513, ZSGB-BIO Co., Ltd., Beijing, China) and DAPI (Sigma, St. Louis, Missouri, USA) were used successively to incubate composites, and the immunofluorescence images were obtained by using confocal microscopy. The fluorescent intensity of COL-2 and proportion of COL-2 positive cells were calculated as previously reported (Wang et al., 2016a; Zhang Z. Z. et al., 2019).

### Cell Morphology on Scaffolds

Cytoskeleton staining was performed, and confocal microscopy was used to observe the morphology of PB-MSCs in hydrogels at day 7. After removal of culture medium of scaffolds and fixing with paraformaldehyde, rhodamine phalloidin (100 nM; Cytoskeleton Inc., Denver, USA) and DAPI working solution (1 μg/mL, Sigma-Aldrich, Inc., USA) stained the cytoskeleton (30 min) and nuclei (5 min) at 37°C, respectively (Wang et al., 2016a).

### Statistical Analyses

All statistical data were expressed as mean ± standard deviation (*SD*). One-way analysis of variance (ANOVA) was used to distinguish the differences among groups after testing for homogeneity of variances, *P* < 0.05 was considered statistically significant. When the results of ANOVA results were significantly different, the Least Significant Difference test (LSD-t) was performed. All analyses were carried out using SPSS 25.0 software (SPSS Inc., Armonk, USA).

## Results and Discussion

### Synthesis of Hydrogel Scaffolds

**Figure 2:**
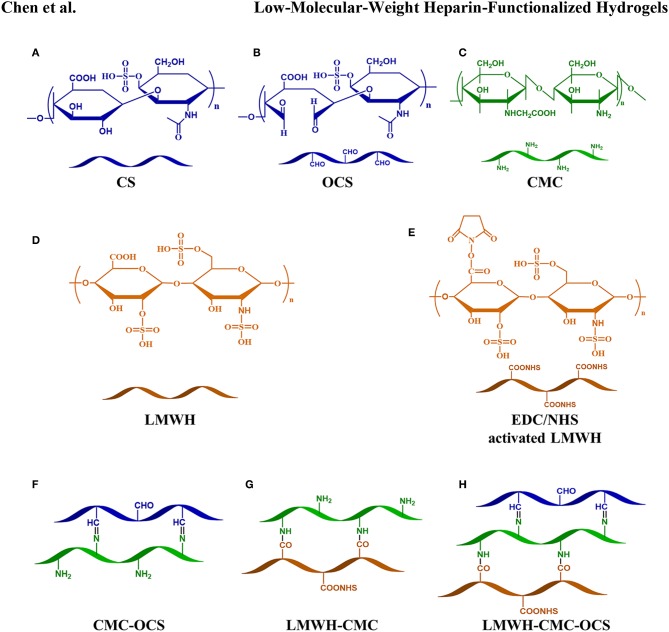
Chemical structures of CS **(A)**, OCS **(B)**, CMC **(C)**, LMWH **(D)**, EDC/NHS activated LMWH **(E)** and schematic diagram of various cross-linked reaction in hydrogels **(F–H)**.

The chemical structures of CMC, CS, OCS, CMC-OCS, LMWH, EDC/NHS activated LMWH and the schematic diagram of various cross-linked reaction in hydrogels were illustrated in Figure 2. OCS was obtained by oxidizing the hydroxyl groups of CS into aldehyde groups using sodium periodate. Also, the carboxylic acid groups (-COOH) of LMWH were activated by reacting with EDC/NHS to obtain activated ester group (-COONHS). The CMC-OCS hydrogel was prepared by simply mixing and the crosslinking network was formed by Schiff base reaction. Moreover, LMWH-OCS-CMC hydrogel was obtained by incorporating the activated LMWH into OCS-CMC hydrogel, wherein the NHS groups of LMWH can chemically linked with amino groups of CMC. In addition, TGF-β3 were *in-situ* encapsulated into CMC-OCS and LMWH-OCS-CMC composites to obtain CMC-OCS-TGF-β3 and LMWH-CMC-OCS-TGF-β3 hydrogels.

### FTIR Spectra of Polysaccharide Derivatives and Cross-Linked Hydrogels

**Figure 3:**
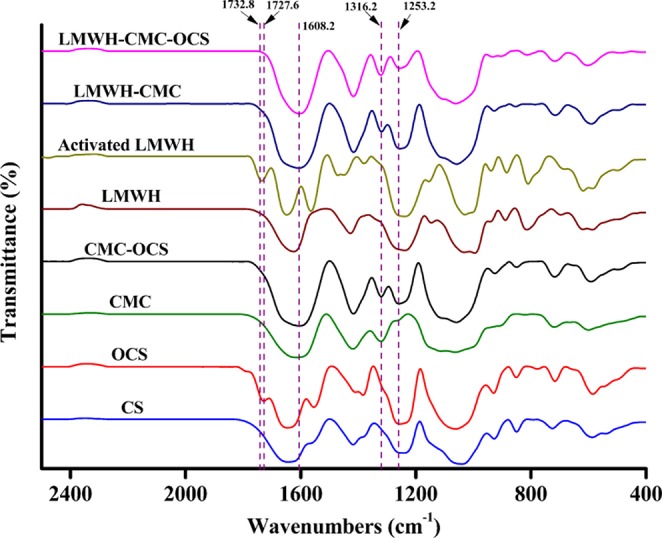
FTIR spectra of polysaccharide derivatives (CS, OCS, CMC, LMWH, and EDC/NHS activated LMWH) and cross-linked CMC-OCS, LMWH-CMC, and LMWH-CMC-OCS hydrogels.

The chemical compositions and structures of polysaccharide derivatives and cross-linked hydrogels were testified by the FTIR in Figure 3. On account of the similar composition of CS and LMWH, they almost exhibited the same characteristic peaks. For example, the peak at 1253.2 cm^−1^ corresponded to S=O stretching vibrations was a characteristic absorption peak of CS and LMWH samples. The peak at 1316.2 cm^−1^ belonged to epoxide ring stretching vibrations was used as a characteristic absorption peak of CMC sample. OCS showed an absorption signal at 1727.6 cm^−1^ corresponded to the aldehyde groups (Fan et al., 2017). By comparing with LMWH, the spectrum of EDC/NHS activated LMWH exhibited a new absorption signal at 1732.8 cm^−1^, which was attributed to the stretching vibrations of C=O of NHS groups (Elahi et al., 2014). For the CMC-OCS hydrogel sample, the coexistence of characteristic absorption peaks of CMC at 1316.2 cm^−1^ and OCS at 1253.2 cm^−1^ and the disappearance of the peak at 1727.6 cm^−1^ revealed that hydrogel cross-linked through Schiff base reaction between CMC and OCS. For the LMWH-CMC sample, the generated characteristic absorption peaks of CMC (1316.2 cm^−1^), LMWH (1253.2 cm^−1^) and the disappeared peak at 1732.8 cm^−1^ demonstrated that EDC/NHS activated LMWH was involved by the formation of amide bonds. The spectra of CMC-OCS, LMWH-CMC, LMWH-CMC-OCS samples were very similar, displaying a absorption signals at 1608.2 cm^−1^ which corresponded to the amide I band (Elahi et al., 2014).

### Morphological Observation of Hydrogels Scaffolds

**Figure 4:**
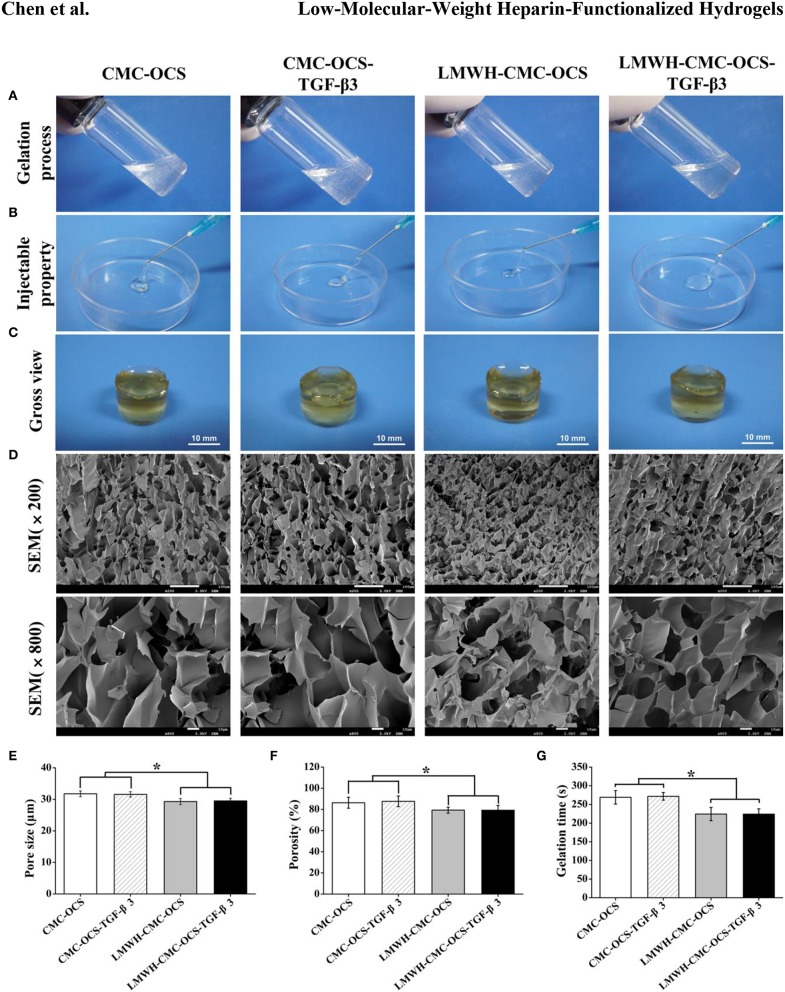
**(A)** Morphologies of hydrogels before gelation. **(B)** The extrusion of four hydrogels from medical syringes to show injectable properties. **(C)** Photographs of gross view. **(D)** Scanning electron micrographs of hydrogels illustrating the porous and interconnected microstructure. **(E)** The average pore sizes of four hydrogels (*n* = 3, ^*^P < 0.05). **(F)** The porosities of four hydrogels (*n* = 5, ^*^P < 0.05). **(G)** Gelation time of four hydrogels (*n* = 5, ^*^P < 0.05).

The CMC, OCS, activated LMWH and TGF-β3 were easily soluble in water and evenly mixed to form a viscous solution. After the extruding and injecting through a conventionally medical syringe, the hydrogels could be generated after several minutes with the colorless and transparence, presenting the injectable property which might be suitable for minimally invasive surgery (Li T. et al., 2018; Chen et al., 2019). After the sufficient cross-linking reaction overnight, the hydrogels were removed from vials and exhibited a clear, slightly brown color as shown in Figures 4A–C. Morphologies of hydrogels were observed in SEM images in Figure 4D, four hydrogels possessed homogeneous porous and interconnected structures. The average pore sizes of CMC-OCS and CMC-OCS-TGF-β3 hydrogels were 31.75 ± 0.91 and 31.58 ± 0.83 μm respectively. After the addition of EDC/NHS activated LMWH, the average pore sizes were decreased to 29.29 ± 0.98 μm (LMWH-CMC-OCS) and 29.49 ± 0.83 μm (LMWH-CMC-OCS-TGF-β3), which may be ascribed to improvement of solid content and crosslinking degree (Figure 4E). Porosities of hydrogels were measured by method of liquid displacement. The porosities of the CMC-OCS, CMC-OCS-TGF-β3, LMWH-CMC-OCS and LMWH-CMC-OCS-TGF-β3 were 86.30 ± 5.16, 87.63 ± 5.03, 79.31 ± 2.80, and 79.21 ± 4.56%, respectively (Figure 4F). The average pore sizes (*n* = 3) and porosities (*n* = 5) of LMWH-CMC-OCS and LMWH-CMC-OCS-TGF-β3 groups were lower than CMC-OCS and CMC-OCS-TGF-β3 groups, with significant difference (^*^P < 0.05). The gelation time of hydrogel was obtained by the vial tilting method in Figure 4G (Bu et al., 2016). The gelation time of CMC-OCS and CMC-OCS-TGF-β3 was 269.11 ± 18.07 and 271.71 ± 10.27 s, respectively. No statistical difference was found between the two groups (*n* = 5, *P* > 0.05). Upon adding the EDC/NHS activated LMWH, the gelation time decrease to 224.24 ± 17.80 and 223.98 ± 14.13 s for LMWH-CMC-OCS and LMWH-CMC-OCS-TGF-β3 hydrogels, respectively. The gelling rate is obviously faster than groups of CMC-OCS and CMC-OCS-TGF-β3 (*n* = 5, ^*^P < 0.05). The gelation time of hydrogels was also determined by the method of rheological measurements. Figure S1 showed that storage modulus G′ of samples increased with time and exceeded dissipative modulus G″, the intersection of G′ and G indicated a gel point. Compared to CMC-OCS and CMC-OCS-TGF-β3 hydrogels, a shorter gelation time was observed in the hydrogels of LMWH-CMC-OCS and LMWH-CMC-OCS-TGF-β3. The faster gelation time was attributed to the much more reactive groups and the higher crosslinking degree in the gelation system.

### Mechanical Properties

**Figure 5:**
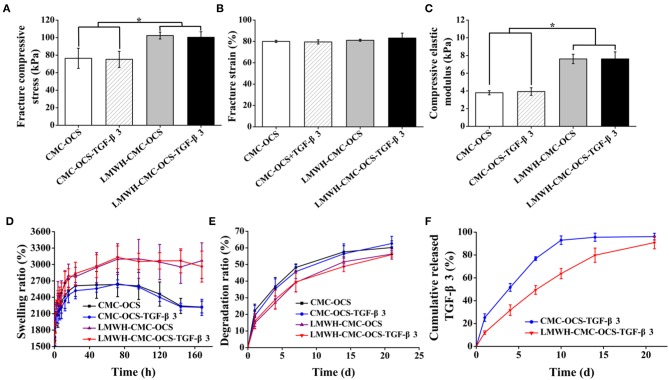
**(A)** Fracture compressive stress (*n* = 3, ^*^P < 0.05), **(B)** fracture strain (*n* = 3, *P* > 0.05), and **(C)** compressive elastic modulus (*n* = 3, ^*^P < 0.05) obtained by unconfined compressive mechanical tests. **(D)** Swelling kinetics of hydrogels in PBS at each time point (*n* = 5). **(E)** *In vitro* degradation of hydrogels in PBS at each time point (*n* = 5). **(F)** Cumulative release of TGF-β3 from CMC-OCS-TGF-β3 and LMWH-CMC-OCS-TGF-β3 hydrogels after incubation for 1, 4, 7, 10, 14, and 21 days (*n* = 5).

Compressive performances of hydrogels were shown in Figures 5A–C. The fracture compressive stresses of CMC-OCS, CMC-OCS-TGF-β3, LMWH-CMC-OCS, and LMWH-CMC-OCS-TGF-β3 hydrogels were 76.44 ± 11.49, 75.23 ± 9.19, 102.41 ± 3.94, and 100.41 ± 6.34 kPa, respectively. The compressive modulus of these hydrogels were 3.80 ± 0.23, 3.93 ± 0.43, 7.61 ± 0.53, and 7.62 ± 0.80 kPa, respectively. There were no statistical differences of the fracture strain (79-83%) among four hydrogels (*n* = 3, *P* > 0.05). The higher compressive properties of LMWH-CMC-OCS and LMWH-CMC-OCS-TGF-β3 hydrogels was due to the denser crosslinking structure and higher solid content compared to CMC-OCS and CMC-OCS-TGF-β3 hydrogels (*n* = 3, ^*^P < 0.05). Oscillatory frequency sweeps experiments were also performed to confirm the mechanical properties of hydrogels (Figure S2). Storage modulus G′ of all hydrogels was higher than dissipative modulus G″, and showed a frequency-independent characteristic. A higher storage modulus G′ was observed in the hydrogels of LMWH-CMC-OCS and LMWH-CMC-OCS-TGF-β3 compared with hydrogels of CMC-OCS and CMC-OCS-TGF-β3, and LMWH-CMC-OCS and LMWH-CMC-OCS-TGF-β3 hydrogels might be more suitable for cartilage repair and regeneration since they were closer to the mechanical strength of native cartilage (Wang et al., 2016b).

### Swelling Property of Hydrogels

The curves of swelling ratio of four hydrogels were illustrated in Figure 5D. All of four hydrogels could reach the swelling equilibrium after incubating at PBS for 72 h. Compared with CMC-OCS and CMC-OCS-TGF-β3 hydrogels, the LMWH-CMC-OCS, and LMWH-CMC-OCS-TGF-β3 hydrogels showed higher swelling ratio (3,100 and 2,600%) and basically maintained their stable swelling behaviors even after 96 h in water, resulting from the higher crosslinking degree of hydrogels. Due to the slight degradation of CMC-OCS and CMC-OCS-TGF-β3 hydrogels after 96 h, the swelling ratio curves displayed a decline trend.

### Degradation Behavior *in vitro*

The degradation behavior of hydrogels was observed in PBS at 37°C. As shown in Figure 5E, the incorporation of LMWH in hydrogels increased their cross-linked density and enhanced the internal structural compactness, therefore the hydrogels containing LMWH showed a slower degradation rate than the hydrogels without LMWH. after incubation for 21 days, the degradation ratio of LMWH-CMC-OCS and LMWH-CMC-OCS-TGF-β3 hydrogels (56.30 ± 3.09 and 55.91 ± 1.40%) was lower than that of CMC-OCS and CMC-OCS-TGF-β3 (60.21 ± 4.04 and 62.62 ± 4.36%). The addition of TGF-β3 into hydrogel had negligible effect on the physical properties of hydrogels. The incorporation of EDC/NHS activated LMWH was beneficial to maintain the structural stability of hydrogel scaffolds and had a significant influence on degradation rate and release rate for a long period of time. Structurally stable hydrogels would facilitate cell adhesion, proliferation and extracellular matrix production *in vitro* (Wang et al., 2019).

### Release of Growth Factors

The release behavior of TGF-β3 was determined by ELISA assay. Figure 5F showed that the release ratio of TGF-β3 from the CMC-OCS hydrogel was quicker than that of functionalized LMWH-CMC-OCS hydrogel. The release ratio of TGF-β3 from CMC-OCS and LMWH-CMC-OCS hydrogel achieve 92.97 ± 3.73 and 63.75 ± 4.50% after 10 days with a sustainable release plateau. The reason was mainly attributed to that the heparin can combine with growth factors (TGF-β3) to form stable complex through electrostatic interaction; in this case, LMWH-CMC-OCS hydrogel displayed a much lower release rate of TGF-β3. In addition, the dense structures and the slow degradation rate of LMWH-CMC-OCS hydrogel may also contribute to the slow continued release behavior with the 90.85 ± 5.48% after 3 weeks. These results confirmed that LMWH incorporated in hydrogel had enough effect on controlling the release of TGF-β3, and that would effectively promote the chondrogenic differentiation of MSCs and induce the expression of cartilage extracellular matrix since TGF-β3 regulated the metabolism of articular cartilage and multifunctional proteins in a time-and concentration-dependent manner (Wang et al., 2017). Therefore, the physicochemical property of hydrogels demonstrated that the LMWH-CMC-OCS hydrogels had shorter gelling time, higher mechanical strength, smaller porosity, higher and more stable swelling ratio, slower degradation rate and lower release rate of growth factors, compared with CMC-OCS hydrogels.

### Isolation, Culture, and Identification of PB-MSCs

**Figure 6:**
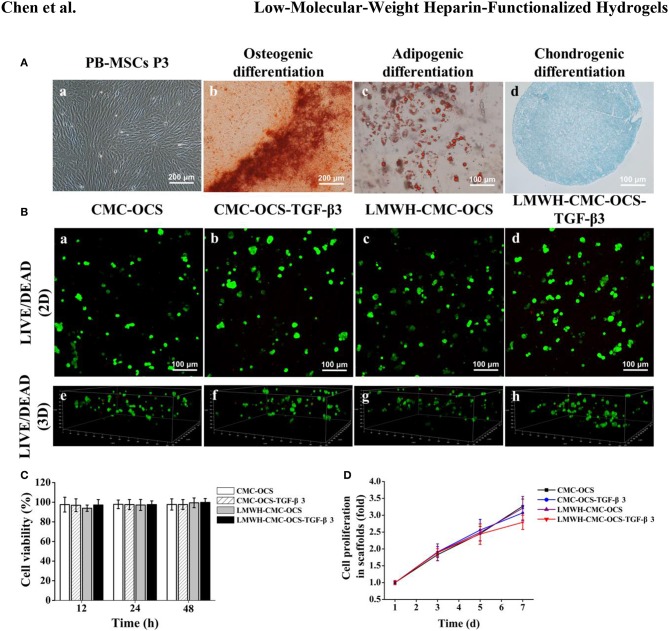
**(A)** The morphological characteristics and multilineage differentiation potential of PB-MSCs. a. PB-MSCs showed a typical spindle shape at passage 3 *in vitro* (×100). b. Osteogenic differentiation was assessed by Alizarin red staining. c. Adipogenic differentiation was detected by Oil Red O staining. d. Chondrogenic differentiation was confirmed by Alcian Blue staining. **(B)** LIVE/DEAD staining of PB-MSCs on various hydrogels. a–d. Cell viability of four groups in hydrogels was demonstrated by using Live/Dead staining after 7 days of culture *in vitro*. Green represented live cells and red represented dead cells. e–h. 3D renderings of PB-MSCs to show distribution of live and dead cells in hydrogels after 7 days of culture. **(C)** Detection of cytotoxicity of hydrogels using CCK-8 assay (*n* = 5, *P* > 0.05). **(D)** CCK-8 assay detected the cell proliferation of four groups over time, the cell proliferation curves of each groups obtained by normalizing the OD value at each point against the average value of the first day.

A number of PB-MSCs clusters began to appear after the culture of initial 12–14 days, and then transformed into typical spindle morphology. After incubation for 21 days, these primary cells achieved the confluence about 80–90%. PB-MSCs exhibited a relative homogeneity at passage 3 (Figure 6A-a).

Multilineage differentiation potential of PB-MSCs was identified with the method of tri-lineage differentiations.

Alizarin red staining was used to detect the deposition of calcium nodules to confirm osteogenic differentiation after 14 days (Figure 6A-b).

Oil red O staining showed the accumulation of lipid vacuoles in cells at 21 days, which suggested adipogenic differentiation of PB-MSCs. (Figure 6A-c). A spherical pellet formed under the condition of micromass culture at 3 days. Alcian blue staining detected cartilage-specific aggregating proteoglycans and determined the chondrogenic differentiation potential of PB-MSCs after incubation for 21 days (Figure 6A-d).

An increasing number of literatures reported that the peripheral blood was a potential alternative source of MSCs and recognized as a similar potential for the proliferation and chondrogenic differentiation as BM-MSCs both *in vitro* and *in vivo* (Fu et al., 2014; Wang et al., 2016a). Based on this, we isolated PB-MSCs with the methods reported above. The PB-MSCs adhered to the bottom of plastic culture dishes and displayed a typical spindle morphology, and differentiated into the osteoblasts, adipocytes and chondroblasts in appropriate conditions *in vitro*.

### LIVE/DEAD Staining of PB-MSCs on Hydrogel Scaffolds

After 7 days of culture in growth medium, LIVE/DEAD Viability Kit assay showed that majority of seeded cells survived in the scaffolds with few dead cells, further confirming the low toxicity of four biocompatible hydrogels (Figure 6B,a–d). Three-dimensional rendering of the cell-scaffolds composites showed that PB-MSCs were evenly distributed on the surface and inside of the hydrogel (Figure 6B,e–h) with the method of centrifugal seeding which could significantly improve the distribution and proliferation of MSCs on scaffolds (Zhang Z. et al., 2015).

### Cytotoxicity of Hydrogel Scaffolds

To detect the cytotoxicity of hydrogels, PB-MSCs were cultured in medium containing extracting liquid of four hydrogels for 12, 24, and 48 h. As shown in Figure 6C, the CCK-8 assay demonstrated that the cell viability could remain over 93%, indicating good biocompatibility of all of the four hydrogels. No statistical differences were found among the four groups (*n* = 5, *P* > 0.05).

### CCK-8 Assay

The CCK-8 assay demonstrated the proliferation of PB-MSCs on four hydrogels during the first week (Figure 6D). There was no statistical difference among the four groups at day 1, 3, and 5 (*n* = 5, *P* > 0.05). However, compared to CMC-OCS and LMWH-CMC-OCS groups, PB-MSCs cultured on CMC-OCS-TGF-β3 and LMWH-CMC-OCS-TGF-β3 hydrogels showed slower proliferation rate at day 7 (*n* = 5, ^*^P < 0.05). It has been reported that the proliferation rate of chondrocytes is slower than that of mesenchymal stem cells (Yang et al., 2018a). Therefore, we concluded that the stronger chondrogenic differentiation of PB-MSCs with these two groups could result in the slower proliferation of PB-MSCs.

### Analysis of Cartilage-Specific and Hypertrophic Genes Expression

**Figure 7:**
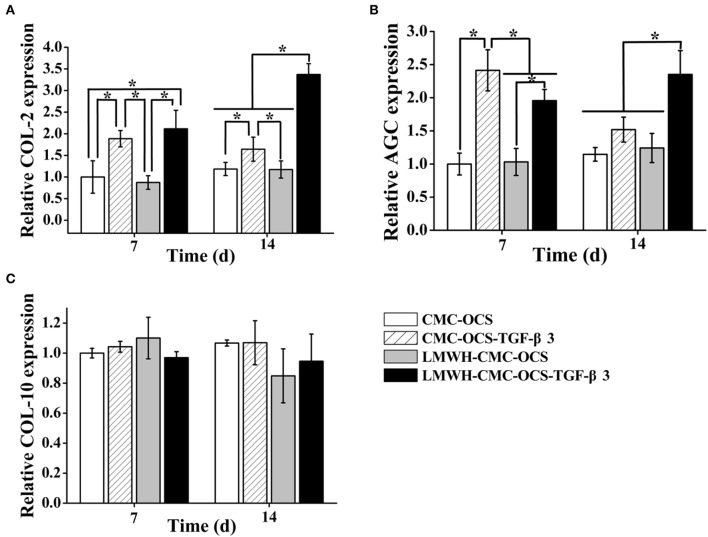
**(A)** Expression of cartilage-specific genes COL-2 and **(B)** AGC (*n* = 3, ^*^P < 0.05). **(C)** Expression of hypertrophic gene COL-10.

We analyzed the expression of cartilage-specific genes COL-2 and AGC, and hypertrophic gene COL-10 (Figure 7). LMWH-CMC-OCS-TGF-β3 showed the highest expression of COL-2 among the four groups at day 7 and 14 (*n* = 3, ^*^P < 0.05). The expression of COL-2 in CMC-OCS-TGF-β3 hydrogel was lower than LMWHH-CMC-OCS-TGF-β3 hydrogel at any time points, although more TGF-β3 released at day 7. We believed that LMWH in hydrogels can protect TGF-β3 from degradation and maintain their biological activity, which might explain the results of the expression of COL-2. AGC expression in the group of CMC-OCS-TGF-β3 peaked at day 7 (*n* = 3, ^*^P < 0.05), but decreased at 14 days. However, the expression of AGC in the hydrogel of LMWH-CMC-OCS-TGF-β3 up-regulated continuously as time extends, and reached its highest expression at day 14 that was obviously higher than the other three hydrogels at any time points (*n* = 3, ^*^P < 0.05). No significant differences of the expression of hypertrophic gene COL-10 was found among the four groups (*n* = 3, *P* > 0.05), and it suggested that four hydrogels could support cell growth and effectively inhibit cell aging.

### Extracellular Matrix Deposition on Hydrogels

**Figure 8:**
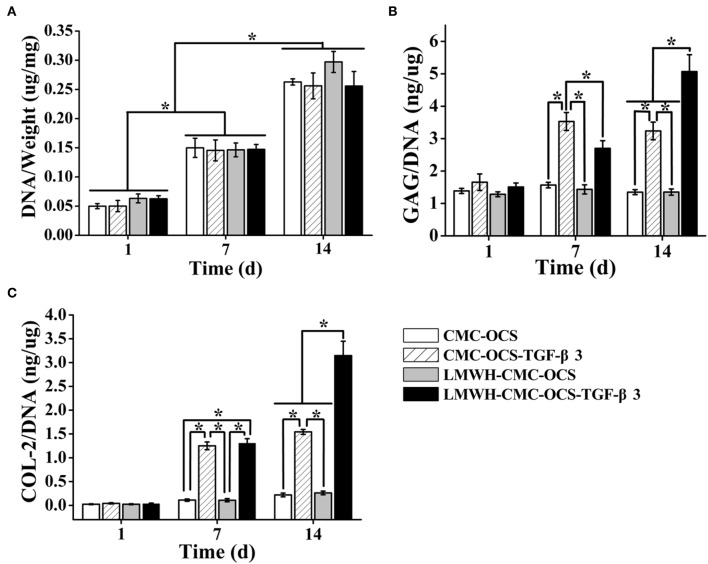
**(A)** DNA contents of four cell-hydrogel composites increased over time (*n* = 3, ^*^P < 0.05). **(B)** GAG and **(C)** COL-2 deposition on four hydrogels by PB-MSCs at different time points (*n* = 3, ^*^P < 0.05).

The DNA contents continued to increase over time during 2 weeks of culture (Figure 8A). The average DNA contents in groups of LMWH-CMC-OCS and LMWH-CMC-OCS-TGF-β3 groups were higher than CMC-OCS and CMC-OCS-TGF-β3 groups at day 1, which was due to the better structural stability and cell adhesion on the LMWH-functionalized hydrogels compared to non-LMWH-functionalized hydrogels. The LMWH-CMC-OCS showed highest average DNA contents at 2 weeks, but no statistical difference was found among four groups (*n* = 3, *P* > 0.05). The contents of GAG and COL-2 in four hydrogels were quantitatively evaluated through ELISA analysis (Figures 8B,C). GAG synthesis in the group of CMC-OCS-TGF-β3 peaked at day 7 (*n* = 3, ^*^P < 0.05), but decreased at day 14. Content of GAG and COL-2 in the hydrogel of LMWH-CMC-OCS-TGF-β3 increased continuously as time extends, and reached its highest amount at day 14 that was obviously higher than the other three hydrogels at any time points (*n* = 3, ^*^P < 0.05). The COL-2 content in the CMC-OCS and LMWH-CMC-OCS groups increased slowly, but the GAG secretion almost remained unchanged.

### Immunoflurorescent Staining of COL-2 and Cell Morphology on Scaffolds

**Figure 9:**
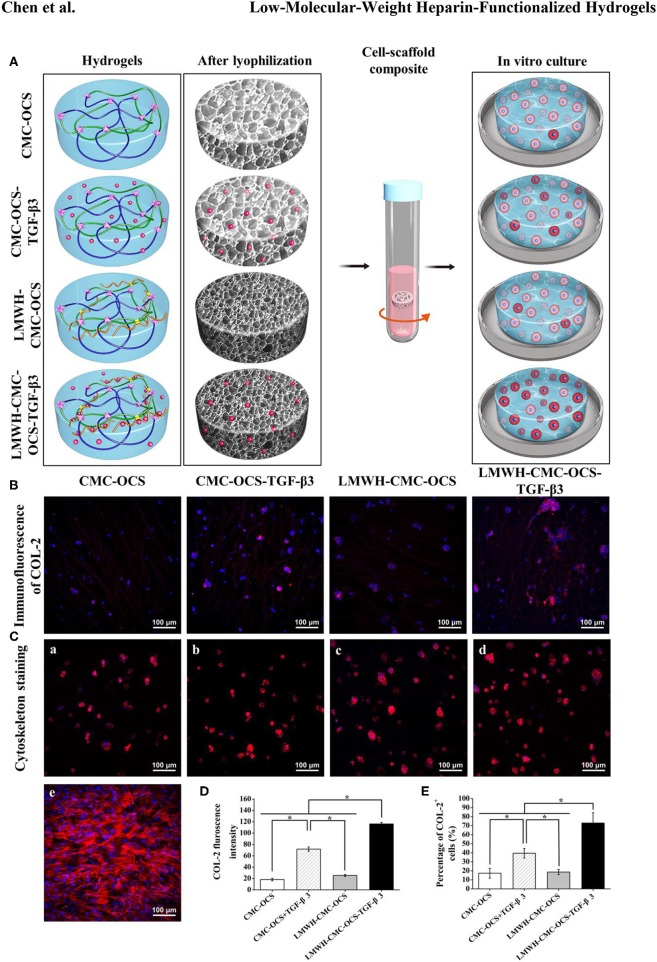
**(A)** Diagrams of four hydrogels before and after freeze-drying, and seeding with PB-MSCs for *in vitro* culture. The redder the cytoplasm represented the higher the degree of chondrogenic differentiation of PB-MSCs in hydrogels. **(B)** Immunoflurorescent staining visualized the production of COL-2 in the PB-MSCs-hydrogel composites after 14 days of *in vitro* culture. **(C)** Cytoskeleton staining revealing morphology of PB-MSCs on hydrogels (a–d) and in culture dish (e) after 7 days of culture. (Red, cytoskeleton; blue, nuclei). **(D)** LMWH-CMC-OCS-TGF-β3 group showed the highest fluorescence intensity of COL-2 (*n* = 3, ^*^P < 0.05) and **(E)** the highest percentage of COL-2 positive cells (*n* = 5, ^*^P < 0.05) among the four groups.

The production of COL-2 protein in cell-hydrogel composites were detected through the immunofluorescent staining after culturing for 2 weeks as shown in Figures 9A,B. COL-2 expression in LMWH-CMC-OCS-TGF-β3 group was stronger than that in the other three groups. In terms of the fluorescence intensity of COL-2 (Figure 9D) and the percentage of COL-2 positive cells (Figure 9E), the LMWH-CMC-OCS-TGF-β3 group showed highest intensity among the four groups (*n* = 3, ^*^P < 0.05). These results of COL-2 immunofluorescence detection were similar to those of ELISA analysis. This is related to the excessive release of TGF-β3 in the early stage and the lack of growth factor in the later stage in the CMC-OCS-TGF-β3 group. Therefore, the long-term slow release of TGF-β3 is more conducive to the expression of cartilage-specific genes and the secretion of extracellular matrix in PB-MSCs, which can be explained by the concentration and time dependence of TGF-β3 on mesenchymal stem cells, and many studies have demonstrated that TGF-β3 stimulate chondrogenesis of mesenchymal stem cells through intracellular pathways involving small mothers against decapentaplegic proteins (Smads) (Chen et al., 2016). As for CMC-OCS scaffold, about 17% of PB-MSCs were positive cells of COL-2 after culturing *in vitro* for 14 days, and COL-2 content also increased as time extended, indicating that CMC-OCS hydrogel could partly promote chondrogenic differentiation of PB-MSCs. The chondroitin sulfate in hydrogels probably contribute to the proliferation and chondrogenic differentiation of seed cells and the synthesis of extracellular matrix (Meghdadi et al., 2019).

Cytoskeleton staining was used to show the morphology of PB-MSCs in four hydrogel scaffolds after culturing for 7 days. PB-MSCs shifted from a spindle-like morphology in the 2D dish (Figure 9C-e) to a similar rounded chondrocyte-like shape in 3D hydrogel scaffolds (Figure 9C,a–d), fully demonstrating the significant effects of CMC-OCS, CMC-OCS-TGF-β3, LMWH-CMC-OCS, and LMWH-CMC-OCS-TGF-β3 hydrogel scaffolds on inducing the chondrogenic differentiation of PB-MSCs.

Although we have constructed a new tissue engineering cartilage scheme with CMC-OCS hydrogel, LMWH controlled-release TGF-β3 and PB-MSCs, there are still some limitations in this study. First, the optimal concentration of LMWH in hydrogel was not further explored. It has been reported that the release rate of growth factor can be regulated by the concentration of heparin in hydrogel. Second, this study was a preliminary research focused on the applicability *in vitro*, the future studies will be conducted on *in vivo*.

## Conclusion

In summary, the LMWH functionalized CMC-OCS hydrogels were facilely prepared through Schiff base and amidation reactions at physiological temperature as biological scaffolds. The hydrogels had highly interconnected porous microstructure with appropriate swelling ratio and degradation ratio, which could favor the ingrowth and proliferation of living cells, as well as water convection. The presence of EDC/NHS activated LMWH within the hydrogel can not only improve the crosslinking degree and enhance the stability of scaffolds in PBS, but also the LMWH can complex the TGF-β3 and realize the long-term controlled release of TGF-β3 by means of electrostatic interaction. *In vitro* experiments proved that PB-MSCs had good tri-lineage differentiation potential and can be used as a suitable seed cell in tissue-engineered cartilage. Live/Dead staining, CCK-8 and extracellular matrix deposition assay displayed that the LMWH-CMC-OCS-TGF-β3 hydrogels had no obvious cytotoxicity and can provide a biocompatible microenvironment for PB-MSCs to growth, chondrogenic differentiation and ECM production. As a new kind of construction strategy for tissue-engineered cartilage, the future studies conducted on *in vivo* are worthy for cartilage repair and regeneration.

## Data Availability Statement

All datasets generated for this study are included in the article/Supplementary Material.

## Ethics Statement

The animal study was reviewed and approved by Animal Care and Use Committee of Peking University Third Hospital.

## Author Contributions

J-KY and Y-YY conceived and designed the study. Y-RC and Z-XZ carried out the experiments, analyzed the data, and drafted the article. J-YZ, F-ZY, and B-BX edited and proofread the manuscript. JG, CH, and DJ provided oversight for this study.

### Conflict of Interest

The authors declare that the research was conducted in the absence of any commercial or financial relationships that could be construed as a potential conflict of interest.
