# Supplementary material for: Low-Molecular-Weight Heparin-Functionalized Chitosan-Chondroitin Sulfate Hydrogels for Controlled Release of TGF-β3 and in vitro Neocartilage Formation
Source: Front Chem. 2019 Nov 1;7:745. doi: 10.3389/fchem.2019.00745 (PMC6839338; doi:10.3389/fchem.2019.00745)
Supplement: Supplementary file 1 [file Table_1.docx]

Supplementary Material

Low-Molecular-Weight Heparin-Functionalized Chitosan-Chondroitin Sulfate Hydrogels for Controlled Release of TGF-β3 and in Vitro Neocartilage Formation

You-Rong Chen^1†^, Zhu-Xing Zhou^1†^, Ji-Ying Zhang^1^, Fu-Zhen Yuan^1^, Bing-Bing Xu^1^, Jian Guan^1^, Chao Han^1,2^, Dong Jiang^1^, Yan-Yu Yang^3,4*^, Jia-Kuo Yu^1*^

^1^Knee Surgery Department of the Institute of Sports Medicine, Peking University Third Hospital, No. 49 North, Garden Road, Haidian, Beijing 100191, China

^2^School of Clinical Medicine, Weifang Medical University, No.7166 West, Baotong street, Weicheng, Weifang 261053, China

^3^Beijing National Laboratory for Molecular Sciences, State Key Laboratory of Polymer Physics & Chemistry, Institute of Chemistry Chinese Academy of Sciences, Beijing 100190, China

^4^College of Materials Science and Engineering, Zhengzhou University, Zhengzhou, Henan 450001, China

† These authors contributed equally to this work.

**Table S1**. Primer sequences used for real-time PCR

| Gene | Forward primers (5'−3') | Reverse primers (5'−3') |
| --- | --- | --- |
| COL-2 | CCACGCTCAAGTCCCTCAAC | AGTCACCGCTCTTCCACTCG |
| AGC | CGTGGTCTGGACAGGTGCTA | GGTTGGGGTAGAGGTAGACG |
| COL-10 | AAGTGGACCGAAAGGAGACA | TGGAAACCCATTCTCACCTC |
| GAPDH | CCATCACCATCTTCCAGGAG | GATGATGACCCTTTTGGCTC |

COL-2: collagen type-2; AGC: aggrecan; COL-10: collagen type-10; GAPDH: glyceraldehyde-3-phosphate dehydrogenase


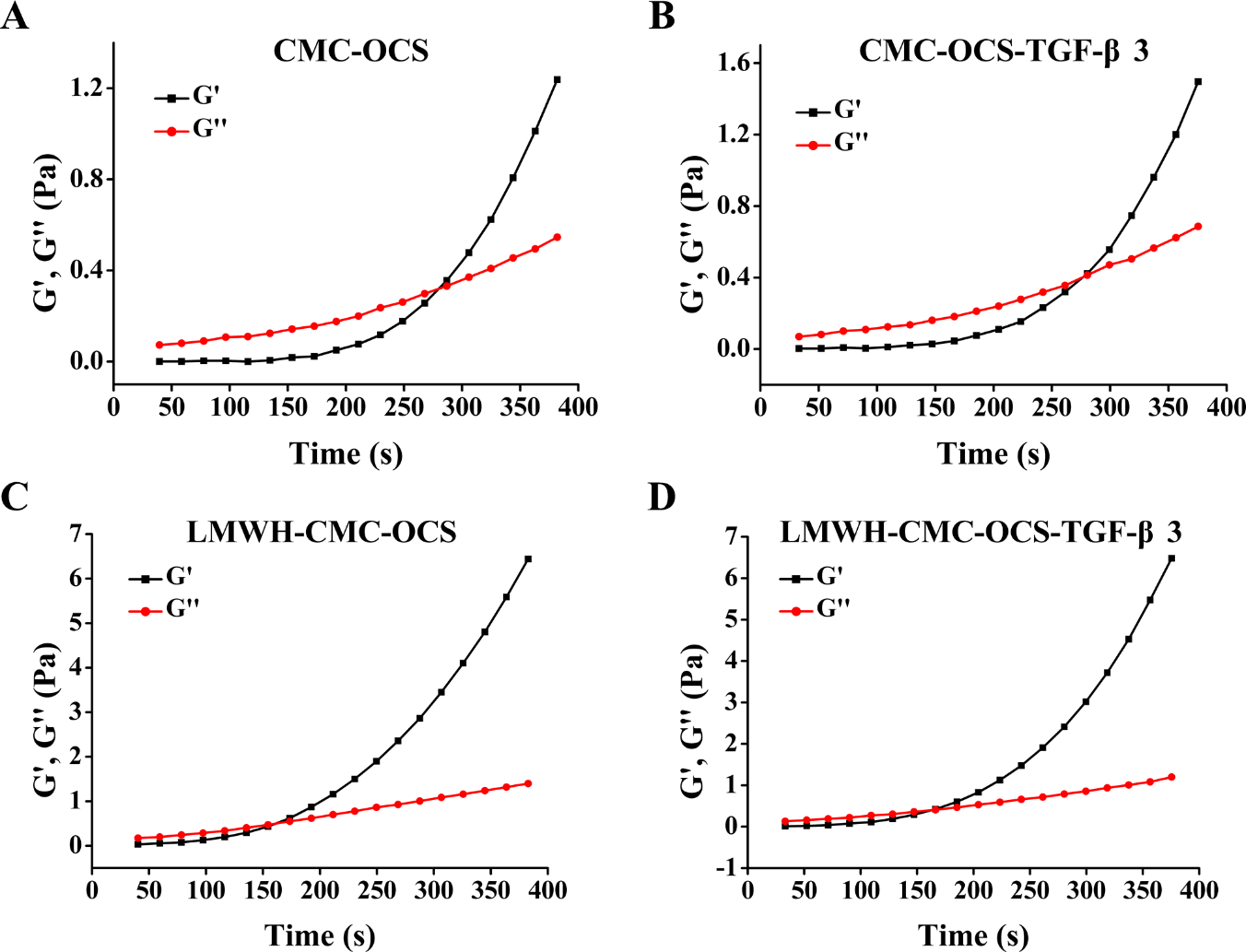


**Figure S1.** Rheological Measurements of time sweeps experiments to determine the gelation time. The time of the crossover point correspond to the gelation time


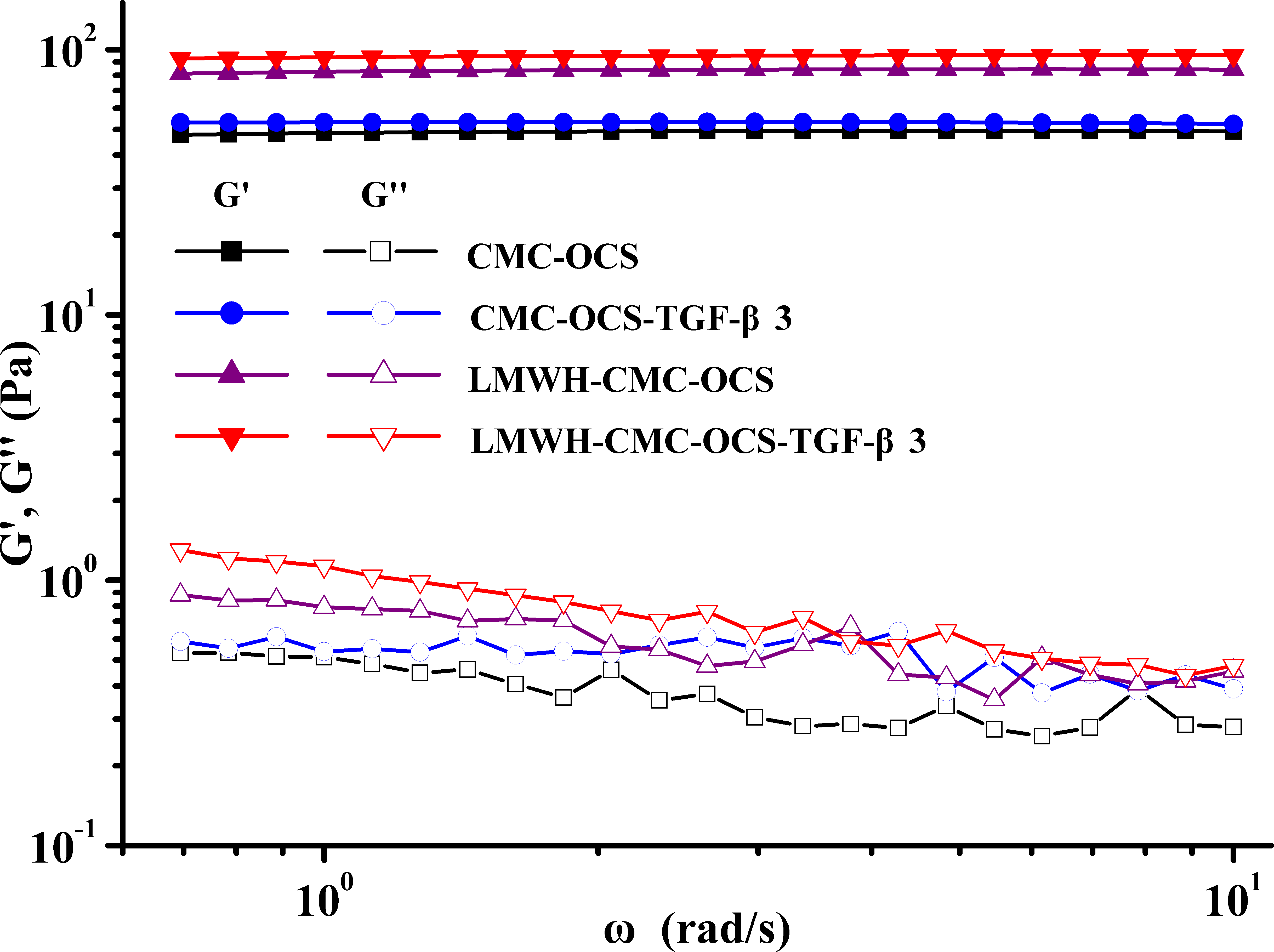


**Figure S2.** Rheological Measurements of oscillatory frequency sweeps experiments used to assess the shear modulus of hydrogels.
